# The Dental Schools

**Published:** 1885-01

**Authors:** 


### EDITORIAL, ETC.

---

THE DENTAL SCHOOLS.—The Session of 1884-'85 of the different dental institutions is drawing to a close, and their Commencement exercises will be held during the latter part of the present and the beginning of the coming month. The present session has been, as far as we have been able to learn, a very prosperous one with some of the schools, while others are in a languishing condition, barely receiving enough to meet the running expenses from the tuition fees of the small number of students in attendance. Such a state of affairs is occasioned in a great measure by the manner in which some of these schools are conducted, the sole motive, apparently, being self-interest without regard to that of the science generally; the least possible sacrifice of time and effort being made by their faculties, and students graduated for their fees only. Another reason for such a deplorable condition is the large number of dental schools, many of them being located in places totally inadequate to furnish clinical material, and some of these schools offering as an inducement to secure students, low fees, which, like cheap dental work, are on a par with what is received by the student. We are gratified to be able to state that the Dental Department of the University of Maryland has had a most prosperous session of 1884-'85, and that the qualifications of the candidates for graduation are such as will reflect honor upon any institution. The specimen mechanical work both of metal and plastic materials, and especially the former is now on exhibition in the Dental Building and open to the inspection of all visitors The contest for the prizes offered to the graduating class will be held on the 14th of March, 1885, and be determined by a committee of dentists not connected with the faculty of the University Dental Department. To this contest all practicing dentists are cordially invited, as the operations performed will demonstrate the efficiency of the contestants.

The commencement exercises will be held at the Academy of Music, Baltimore, March 17th, 1885, in connection with the Seventy-ninth annual Commencement exercises of the Medical Department. One of the most gratifying facts connected with the Dental Department of the University of Maryland is, that, so far as we know, no student who has ever attended one course at this institution, has gone elsewhere to graduate.

